# PDI-Regulated Disulfide Bond Formation in Protein Folding and Biomolecular Assembly

**DOI:** 10.3390/molecules26010171

**Published:** 2020-12-31

**Authors:** Jiahui Fu, Jihui Gao, Zhongxin Liang, Dong Yang

**Affiliations:** Beijing Key Laboratory of Functional Food from Plant Resources, College of Food Science & Nutritional Engineering, China Agricultural University, 17 East Tsinghua Rd., Beijing 100083, China; s20193060969@cau.edu.cn (J.F.); jhgao@cau.edu.cn (J.G.); SY20193061085@cau.edu.cn (Z.L.)

**Keywords:** PDI, protein folding, disulfide bond, gluten network, dough rheology

## Abstract

Disulfide bonds play a pivotal role in maintaining the natural structures of proteins to ensure their performance of normal biological functions. Moreover, biological molecular assembly, such as the gluten network, is also largely dependent on the intermolecular crosslinking via disulfide bonds. In eukaryotes, the formation and rearrangement of most intra- and intermolecular disulfide bonds in the endoplasmic reticulum (ER) are mediated by protein disulfide isomerases (PDIs), which consist of multiple thioredoxin-like domains. These domains assist correct folding of proteins, as well as effectively prevent the aggregation of misfolded ones. Protein misfolding often leads to the formation of pathological protein aggregations that cause many diseases. On the other hand, glutenin aggregation and subsequent crosslinking are required for the formation of a rheologically dominating gluten network. Herein, the mechanism of PDI-regulated disulfide bond formation is important for understanding not only protein folding and associated diseases, but also the formation of functional biomolecular assembly. This review systematically illustrated the process of human protein disulfide isomerase (hPDI) mediated disulfide bond formation and complemented this with the current mechanism of wheat protein disulfide isomerase (wPDI) catalyzed formation of gluten networks.

## 1. Introduction

The earliest concept of “protein folding” was raised by Christian B. Anfinsen, who considered that all information about the three-dimensional structure of a protein is stored in its amino acid sequence, e.g., a protein could fold itself correctly without external help or impact [1]. Later on, John Ellis proposed a new notion of protein folding: the folding and assembly of a certain polypeptide chain to form the correct oligomeric structure is ensured by proteins that act as molecular chaperones [2]. Molecular chaperones are a large and diverse group of proteins that share the capability of assisting noncovalent folding and unfolding, the assembly and disassembly of other macromolecular structures, while they would not become a permanent part of these structures after finishing their duties [3]. The theory of molecular chaperones-assisted self-assembly does not necessarily conflict with the thermodynamic hypothesis established by Anfinsen, but rather expands the protein folding theory from a kinetic perspective [1].

Oxidative protein folding is a special protein-folding process accompanied by the formation of disulfide bond(s). It may be the most complicated protein folding scenario, due to the possibility of protein misfolding being increased with increasing cysteine numbers in a protein, where only one disulfide bond pattern is proper for the correct folding [4]. Moreover, redox-active molecules, such as glutathione (GSH), oxidized glutathione (GSSG), thiol compounds, and so on, also exhibit significant effects on the rates of oxidative protein folding and the yield of native proteins [5]. In all cases, the process of generating a natural protein conformation involves both disulfide bonds formation and isomerization steps [6]. Disulfide bonds, which stabilize protein conformation and participate in the regulation of the protein redox process, are very important for the structure, function, and regulation of protein activities. In addition, the formation of disulfide bonds is a key rate-limiting step in protein folding, and the efficiency of spontaneous disulfide bond formation is far lower than that under enzymatic catalysis. It′s worth noting that misfolding occurs when cysteine residues are mismatched into disulfides [7]. Misfolding of protein often leads to the loss of its biological function and the emergence of many diseases, such as Alzheimer′s disease, Parkinson′s disease, and type II diabetes [8,9,10,11]. However, organisms utilize many mechanisms, including isomerases and enzymes that degrade misfolded proteins, to maintain the protein homeostasis [12]. In other words, correct protein folding in vivo is achieved under the efforts of many enzymes and chaperones.

The classic substrate, ribonuclease A, was firstly used to study the oxidative folding catalyzed by enzymes, and since then the key catalyst—protein disulfide isomerase (PDI)—has been extensively characterized [13,14,15]. For more than half a century′s study, researchers have revealed the physiological functions, biochemical properties, and three-dimensional structures of PDI, and it was shown to play a critical role in the oxidative protein folding. PDI catalyzes the oxidative folding of many substrates with well-defined oxidative folding pathways. For example, in previous studies, researchers demonstrated that PDI specifically recognized folding intermediates, such as N* and N′, in bovine pancreatic trypsin inhibitors, to effectively guide their oxidative folding [16,17,18,19]. PDI-catalyzed oxidative protein folding is virtually a process in which enzymes interact with substrates and catalyze the formation of native disulfide bonds in the substrate protein. To effectively guide a proper oxidative protein folding, it is believed that PDI regulates its conformational and oligomeric states’ dynamics according to its redox states and the folding states of its substrates [20,21]. In this paper, we reviewed the mechanism of human protein disulfide isomerase (hPDI) catalyzed oxidative protein folding and the mechanism of wPDI-catalyzed glutenin macropolymers (GMP) formation, for a timely update of PDI studies.

## 2. Conservative Primary and Dynamic Tertiary Structures of hPDI

**Figure 1:**
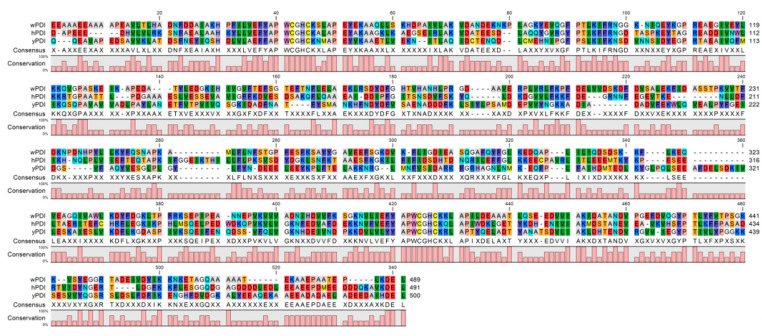
Sequence alignment of wheat protein disulfide isomerase (wPDI) [37], human PDI (hPDI, NCBI: NP_000909.2), and yeast PDI (yPDI, NCBI:P17967.2). Red indicates amino acid residues D, E; yellow indicates C, M; blue indicates R, K; violet indicates F, Y; dark green indicates I, L, V; cyan indicates Q, N; light grey indicates A; tawny indicates P; orange indicates S, T; mauve indicates W; white indicates G; pale blue indicates H.

PDI is ubiquitous in organisms. Structural and functional studies of protein disulfide isomerases (PDIs) from various species, including bacteria, wheat, humans, and yeast etc., have been reported after it was identified as an enzyme that facilitates protein folding [13,22,23,24,25,26,27,28]. Meiri et al. categorized 51 PDI-like proteins, which contained four types key domains (a, b, c, and Erp29c), into six clusters based on their sequences [29,30]. Most mammalian PDIs, but not all, belong to cluster six according to this classification, owing to the fact that those PDIs possess four typical domains (a, b, b′, and a′). For example, ERp72 of humans has five thioredoxin-like domains and thus belongs to cluster three [31]. Among mammalian PDIs, either the functional mechanism or structure of hPDI has been intensively studied [24]. Currently, the hPDI family has more than 20 members, and each of them consists different numbers of thioredoxin-like domains, which usually contribute to the oxidative folding and disulfide bond rearrangement in substrate proteins [32,33]. As for higher plant PDIs, wheat PDI (wPDI) is discussed here. There are nine PDI genes that have been identified, and those PDIs and PDI-like wheat genes show high diversity in their genomic organization, sequences, and domain constructs of their deduced proteins. Interestingly, each wPDI gene shows high structural conservation with the genes from other plant species in the same phylogenetic group [34]. On the other hand, the constructs of hPDI family members differ from that of higher plants and yeast [35,36]. Here, we chose several PDIs from three different species, including protein disulfide isomerase from wheat (wPDI), human (hPDI), and yeast (yPDI), and compared their amino acid sequences (Figure 1). The sequence of hPDI exhibited high degree (40.80%) of sequence homology when compared with wPDI and yPDI. Meanwhile, there was a highly conserved catalytic region (EFYAPWCGHCK) maintained close to the N- and C-terminuses, and an endoplasmic reticulum (ER) retention signal sequence at their C-terminuses. These above PDIs all contain four thioredoxin-like domains with a βαβαβαββα structure.

Generally, hPDI contains four distinct structural domains (a, b, b′, and a′) deduced from its tertiary structure, plus an inter-domain region, named x-linker, between the b′ and a′ domains and a c tail [38,39]. It is an elongated monomer where its domains are arranged almost linearly as a, b, b′, x, a′. The catalytic a and a′ domains are homologous to thioredoxin, and each contains a catalytic CXXC motif, which is essential to provide efficiently the donor of reducing equivalents for the formation of substrate protein disulfides. These active-site cysteine residues can either be in the form of free sulfhydryl or form a pair of disulfide bonds with each other. The active sites of PDI exhibit high reduction potentials that mainly depend on the pKa values of the two cysteine residues [40]. PDI′s redox potential affects the ratio of reduced PDI [41]. The physiological functions of hPDI, including oxidation, reduction, and isomerization, are determined by the redox equilibrium in the catalytic sites under cellular conditions. So, not only the pKa value, but also their redox potential, is of importance for the physiological functions of hPDI [36]. Besides, the enzymatic activity of PDI is affected by the X amino acid in the active site CXXC sequence [42]. The b′ and b domains, which are not the catalytic sites, are also homologous to the theoredoxin domains [38,43]. Studies have shown that the role of the b′ domain is to bind substrate proteins [44,45,46]. The c tail contains many acidic amino acids and an ER retention signal [47,48]. Structural and biochemical studies indicated that the a′ and b′ domains are primarily responsible for substrate recognition. In particular, mutational and cross-linking analyses found that the b′ domain provides the principal substrate-binding site, whereas the a′ domain acts as a disulfide donor/receptor for the substrates during the redox process [49,50].

To effectively guide proper oxidative protein folding, the conformation of hPDI changes according to its redox state [24,51,52]. Solid evidences concerning the redox-regulated conformational changes of hPDI have been supported by several studies [20,24,53]. For reduced PDI, it is considered as a “closed” conformation since it limits the binding of substrate for both human and fungal PDI [23,52,54]. For oxidized hPDI, the cysteine residues in the active site are connected by disulfide bonds, which are considered an “open” conformation. The hydrophobic surface of the a′ and b′ domain is exposed when reduced hPDI is transited to oxidized hPDI, providing a capability for binding extended/unfolded proteins or less-folded intermediates [51,55]. The conformational changes during the redox cycles are mainly located in the b′xa′ region by comparing structures of hPDI in different redox states [24]. Particularly, once the redox state of the a′ domain in fungal PDI changes, the b′xa′ domains undergoes conformational rearrangement, and therefore the x region moves away from a conformation in which it caps the substrate binding site [55,56]. Change in the conformation of the x region between the ′′capped′′ and ′′uncapped′′ states also results in a significant movement of the a′ domain with respect to the b′ domain [15]. Recently, studies have found that the stability of hPDI conformation, which is both redox-dependent and domain-specific, will increase in the absence of a′ domain [53]. Thus, the redox-regulated conformational transition is supposed to be related to the substrate protein binding.

In addition to its redox-sensitive structural change, hPDI also exhibits substantial conformational flexibility to substrate. Many structural studies have identified that hPDI is highly flexible in the substrate binding and release cycles [57,58,59]. Generally, the ab domains are more flexible than the bb′ domains during hPDI redox cycle. However, the most flexible region is the b′xa′ region, especially the a′ domain, which changes greatly in hPDI′s redox transition [51]. Moreover, several researchers have discovered that hPDI contains multiple binding sites with moderate to low affinities, which allows hPDI to accommodate a wide variety of substrates with different conformations and sizes or at different folding extents, and promotes the release of the substrate from hPDI when the reaction cycle is completed [22,60,61]. These prominent structural features enable hPDI to exert its biology function as an oxidoreductase and/or a chaperone efficiently. Crystal structures of PDI from humans and yeast have revealed that the substrate binding site is partially occupied by another PDI molecule [24,62,63]. This is probably due to the fact that PDI stabilizes its conformation by binding to another PDI molecule under crystallization conditions.

## 3. Biochemical Activities of hPDI

ER is the primary site where hPDI performed its activities in vivo, and hPDI is reckoned as a crucial component of the ER protein folding capacity as it mediates the disulfide bond formation during protein folding. When the misfolded or unfolded proteins accumulate within the ER, they give rise to ER stress. ER stress subsequently activates the unfolded protein response (UPR), which aims to alleviate the ER stress. In UPR, an important mark is the upregulation of protein chaperones to improve the ER folding capacity. hPDI is one of these chaperones, and it not only improves the folding level of misfolded proteins when UPR occurs, but also performs these activities in normal cell conditions. hPDI primarily achieves its function of catalyzing protein by performing the following biochemical activities: oxidation, reduction, and isomerization of disulfide bonds in substrate proteins in ER [36,48,64].

### 3.1. Oxidoreductase Activity

To achieve the folding of a native protein, PDI catalyzes repeated reaction cycles of dithiols to disulfides, which is mainly related to the oxidoreductase activity of PDI. Catalysis by PDI as an oxidant can be attributed to its high reactivity of the active site [65]. Specifically, the minimal unit is considered to be an isolated a or a′ domain from hPDI, which is able to catalyze the oxidation reactions efficiently in substrate proteins [66,67]. hPDI exerts oxidase activity, in which the disulfide bond in the active site is “transferred” to the substrate proteins while hPDI itself is reduced, in which cysteines in the active site are free sulfhydryl. GSSG has been regarded as the oxidant of hPDI in the ER. It is believed that GSSG acts as an electron acceptor, oxidizing sulfhydryl groups in the active site of hPDI and converting them into disulfide bond again [68]. It was not until the discovery of the flavoprotein, which was also called endoplasmic reticulum oxidoreductase 1 (Ero1), in ER, and the identification of its biological functions that the above theory was implemented. It is believed that PDI is the physiological substrate of mammalian Ero1, and the oxidation of PDI is mainly under the assistance of Ero1 [69]. However, the oxidation of plant PDI may also require the assistance of plant Ero1 [70,71].

Complementary to its oxidase activity, hPDI also exhibits reductase activity, which converts cysteines in the active site of hPDI into a disulfide bond, while the substrate protein is reduced. An electron donor, such as dithiothreitol, is required to reduce the disulfide bond at the active site of hPDI for completing its catalytic cycle. In essence, the reductase efficiency of PDI is much lower than that of the thioredoxin reductase, indicating that the reductase activity is not the primary catalytic function of PDI [72].

### 3.2. Isomerase Activity

Any two cysteine residues in a protein have the potential to form a disulfide bond, except those too adjacent ones. The more cysteine residues in a protein, the higher the possibility of forming disulfide bonds. Nevertheless, a protein usually needs only one correct mode of disulfide bond(s) and free thiol(s) in its natural state. Many proteins presumably form both native and nonnative disulfide bonds during oxidation. Cysteines are prone to mispairing in the early stages of protein folding, resulting in failure to reach their native structures [73]. Once incorrect disulfides are formed, disulfide isomerization is required to convert the disulfides to their native conformation, where the isomerase activity of PDI is often employed.

Protein isomerization reaction catalyzed by PDI contains two mechanisms, namely the direct isomerization mechanism in the substrate molecule and the “reduction/oxidation cycle” isomerism mechanism [48]. The former refers to the reaction where the active site cysteines near the N-terminus perform a nucleophilic attack on the substrate protein. When the cysteine residues at the N-terminal attack the disulfide bond in the substrate to form a PDI-substrate covalent complex, the reaction that the released sulfhydryl groups (SH) in the substrate decides the “destiny” of the complex. There is no isomerization if the active site of PDI from rat reversibly attacks the disulfide linkage in PDI-substrate, and PDI is released without disrupting the disulfide bonds of substrate [74]. Since no net change exists in the redox state of PDI′s active site, no other redox equivalents are needed in the direct isomerization mechanism [15]. This simple reversible reaction mechanism may endow the function of hPDI recognizing natural or mismatched disulfide bonds [75]. Additionally, when a sulfhydryl in the substrate attacks the disulfide bond in the complex and replaces the cysteine in PDI, an isomerization process occurs through an intramolecular disulfide bond rearrangement within the substrate itself. In particular, rat PDI still retains part of its isomerase activity even if the cysteine at the C-terminal active site is mutated to serine (CGHS), indicating that the active-site cysteine in the C-terminal has limited impact on the direct intramolecular isomerization [74].

Although cysteine at the C-terminal active site of PDI is not essential for the direct isomerization reaction, it does participate in the “reduction/oxidation cycle” isomerization mechanism. Studies have reported that cysteine mutants in C-terminal active sites still retain some isomerase activity and cycling of redox reactions can be observed during isomerization [76,77]. In this isomerization mechanism, the cysteine residues at the C-terminal active site attack the disulfide bond of the PDI-substrate complex, where the disulfide bonds of the substrate protein are reduced to free sulfhydryl, which is regenerated by oxidized PDI, during which the conformational changes occur. Meanwhile, PDI is separated from the complex in the form of its oxidized state. In this way, isomerization can be viewed as repeated cycles of reduction and re-oxidation, leading to disulfide rearrangements until the configuration of substrate is correct [74].

### 3.3. Chaperone Activity

In addition to the above enzymatic activity, hPDI also exhibits molecular chaperone activity, through which it stabilizes the unstable conformation of substrates and promotes the folding of nascent polypeptide chains [78,79]. Thus, it is widely accepted that hPDI is both an enzyme and a chaperone [80]. It is difficult to distinguish the chaperone activity of hPDI from its other enzymatic activity because the folding of protein and the formation of disulfide bonds are two inseparable, while coordinated, processes. To explore the chaperone activity of mammalian PDI explicitly, proteins without disulfide bonds, such as glyceraldehyde phosphate dehydrogenase(GAPDH) or rhodanase, were used as the substrate proteins [81,82]. The accelerated folding by bovine PDI cannot be attributed to its enzymatic activity but only to its chaperone activity due to the fact that reactivation of these target proteins has nothing to do with the formation of disulfide bonds. Results showed that PDI is not part of the final functional unity when bovine PDI refolds the denatured GAPDH or rhodanase. This is in line with the molecular chaperone criteria [83].

PDI is a molecular chaperone with low specificity that binds to peptides with various amino acid sequences, lengths, or charge distributions. Studies on the crystal structures of the truncated hPDI protein bb′a′ domains revealed the molecular mechanism of the chaperone activity of hPDI is regulated by the redox state of cysteine at the catalytic site of the a′ domain [23,84]. The hPDI molecular chaperone activity is enhanced when its active site in the a′ domain maintains its oxidized state. However, the chaperone activity of rabbit PDI, which is regulated by the redox state of the active site of the a′ domain, does not depend on the cysteine residue in this active site [85]. After chemically modifying the cysteine residues in the active site of hPDI, other enzymatic activity of modified hPDI is lost, while the molecular chaperone activity is retained. Furthermore, hPDI that lost some of its enzymatic activity still plays a crucial role in organisms [84,86]. Research has indicated that a PDI variant still maintains 5% of its wild-type PDI isomerase activity when the CGHC motif in the PDI active site has been mutated into CLHS or CIHS, and this variant with both CGHC active sites replaced by SGHC has abolished its isomerase activity but still accelerates protein folding and secretion in the cell of humans or yeast [86,87]. Recently, Rosenberg and colleagues also indicated that hPDI enhances cell adhesion by both its oxidoreductase and chaperone activity [88]. These experiments unambiguously discriminated the chaperone and oxidoreductase activities of hPDI in the oxidative folding of disulfide-containing proteins, and demonstrated that both activities are necessary for hPDI to function as an efficient folding catalyst [89]. Actually, the chaperone function of mammalian PDI is complementary to its oxidoreductase activity, though they are distinct [85,90]. The chaperone activity of hPDI is dependent on its conformation, and the latter is coupled to its redox state [51]. Oxidation of hPDI results in the self-conversion from the compact conformation to the open conformation with the substrate-binding surface more exposed, exhibiting higher chaperone activity to prevent the aggregation during substrate refolding [23].

## 4. Substrate Binding and Domain Coordination in hPDI

The role of each PDI domain on its activity has been determined by assaying the PDI constructs with deletion and domain rearrangement. It is now known that each hPDI domain has its own function and interacts with others during the process of substrate binding. The a′ domain exhibits a high degree of flexibility that allows the U-shaped cleft between hPDI domains to accommodate substrate proteins with various sizes. Substrate oxidation only requires the participation of a or a′ domain, while the isomerization requires the coordination between a or a′ and b′ domains. Furthermore, isomerization reactions involving extensive conformational changes of the substrate require all domains of hPDI except the c tail, suggesting that the domains of hPDI are able to coordinate with each other and they jointly stabilize a partially unfolded conformation of the substrate. Studies on several proteins with clear folding paths confirmed these aforementioned functions of the domains [66,91,92]. The work of Klappa and coworkers has shown that substrates can only be chemically cross-linked with PDI containing the b′ domain and that the b′ construct of hPDI alone is sufficient to bind small peptides [44,93]. Certainly, to bind larger proteins, the b′a′c domains are required, indicating that the b′ domain acts as the principal substrate binding site and interaction with other domains facilitates binding of larger substrates [44]. It is likely that the b′ domain, in some cases together with other domains of hPDI, interacts with the side chains and backbone regions of substrate exposed in a partially unfolded conformation. Meanwhile, the catalytic sites of the a and a′ domains are contributing to the binding of misfolded proteins and participate in thiol–disulfide exchange [15,36]. The thiol–disulfide exchange reactions catalyzed by hPDI are not only essential for maintaining proper redox state of thiols in proteins, but also indispensable for boosting the formation of disulfide bonds within substrates. What is more, binding of substrate to the b′ domain is sensitive to the conformational changes of other domains. Therefore, changes in other domains of hPDI, such as mutations in the a′ domain, indirectly affect the b′ domain in accommodating its client proteins [94]. This may be on account of the fact that the mutation has altered the conformational balance in the hPDI, where the substrate-binding site in the b′ domain is covered by the x linker, thereby being prevented from accommodating the substrate proteins. Generally, at least three domains of PDI are involved in its interaction with substrates [58].

## 5. Catalysis Mechanism and Redox Regeneration of hPDI

**Figure 2:**
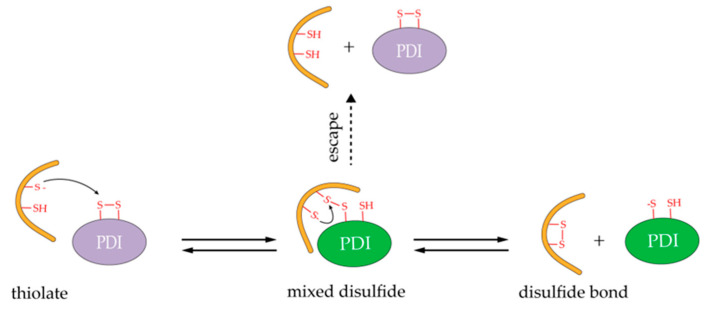
Schematic of sulfhydryl groups (SH)/(disulfides) SS exchange reaction catalyzed by PDI. Only one active site of PDI is shown for simplicity (similarly hereinafter). Yellow represents substrate proteins; red represents free sulfhydryl, disulfide bond, or thiolate; purple represents oxidized PDI; green represents reduced PDI.

Bulaj believed that disulfide bonds formation is a reversible process and requires two steps to complete [95], as illustrated in Figure 2. The first step is that thiolate provided by substrate directly launches a nucleophilic attack on the disulfide of catalyst, such as PDI, leading to the formation of a mixed disulfide (substrate–PDI) intermediate. Then, the mixed disulfide species is broken by another nucleophilic attack from an additional substrate cysteine thiolate. As a result, PDI is released from the mixed disulfide and a disulfide bond in the substrate protein is formed. Moreover, additional thiol in PDI provides an escape pathway if the interchange reaction is slow (Figure 2) [41,96]. It should be noted that the second nucleophilic attack may come from the substrate itself (intramolecular thiolates) or from another protein’s molecule (intermolecular thiolates), which is determined by the “effective” concentrations of the cysteine pairs and the total protein concentration in the folding solution [95]. In vitro studies have shown that the effective K_m_ value of PDI for peptide substrate is less than 3 μM and the effective concentration of sulfhydryl in unfolded substrate generally ranges from 1 to 100 mM [95,97]. This means that very dilute concentrations of proteins are needed during the oxidative folding reaction.

**Figure 3:**
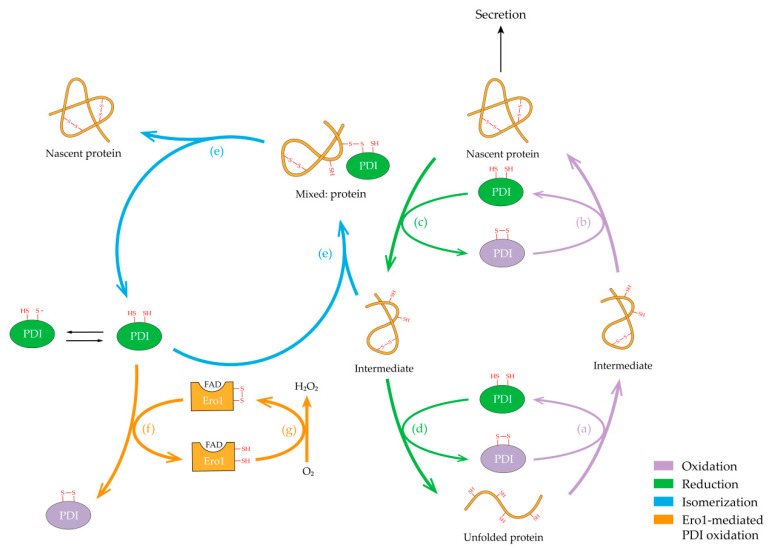
The pathway of PDI catalyzed disulfide formation and isomerization in the ER. Unfolded protein is catalyzed by oxidized PDI, resulting in the formation of intermediate (**a**), which can further be oxidized to generate nascent protein possessing biological functions (**b**). Reduced PDI catalyzes the reduction of disulfide bonds in nascent peptide to facilitate substrate unfolding (**c**), and form an unfolded protein eventually (**d**). It is worth noting that the oxidation/reduction pathway involves repeated cycles of oxidized/reduced PDI. PDI would carry out the isomerization pathway, in which a mixed substrate protein is generated, if the intermediate is misfolded (**e**). After substrate thiol oxidation, oxidized PDI can be regenerated though an alternative pathway (**f**). Endoplasmic reticulum oxidoreductase 1 (Ero1) containing flavin adenine dinucleotide (FAD), which uses O_2_ to reoxidize itself for further folding cycles (**g**), plays a dominating role in this way. Mauve indicates the oxidation process, green indicates the reduction process, blue indicates the isomerization process, orange indicates the Ero1-mediated PDI oxidation process.

Typical, mammalian PDI, such as hPDI, contains two conserved catalytic domains, which can either form an intramolecular disulfide (oxidized hPDI) or exist in the dithiol form (reduced hPDI). The reaction that PDI catalyzes depends on the equilibrium position of the overall reaction and the redox state of PDI active site [48]. Depending on the redox states of its active sites, PDI catalyzes disulfide bond formation and isomerization in proteins involving a series of thiol/disulfide exchange reaction (oxidation/reduction) between cysteine thiolates and an oxidizing disulfide, and the rearrangement of disulfide bonds (isomerization) in the substrate (Figure 3) [51]. The cysteine residues in the active site close to the N-terminus of the PDI activate the disulfide bond formation mechanism by attacking the substrate disulfide bond. This nucleophilic cysteine is exposed to solvent and reacts with substrates, while the other active site is buried and reacts only with the nucleophilic cysteine [74]. The covalent intermediate generated by the PDI attacking the substrate disulfide bond yields three possibilities. First, the oxidized PDI continues to attack the complex between PDI and substrate so that it is further oxidized to produce its natural conformation. Subsequently, the nascent protein could be secreted out of ER to perform its biological functions. The second possibility is that the substrate protein is reduced due to the attack of a reduced PDI. In these cases, isomerization reaction could not occur because the disulfide bonds of the substrate are already paired properly. Nevertheless, once an incorrect substrate disulfide forms, isomerization is required to convert the disulfides to their native arrangement, which requires breaking the wrong cysteines connected and reforming the disulfide bond in substrate (Figure 3). Isomerization can be viewed as repeated cycles of redox reaction, leading to continuous rearrangement of disulfide bonds until the configuration of the substrate is correct. Interestingly, if the PDI-catalyzed native disulfide bonds form with a higher rate than those unnatural ones, the reversibility of the initial reaction step provides PDI with a mechanism that distinguishes native disulfides from incorrect ones [48]. What is more, complicated substrate may lead to a slow arrangement of protein disulfide bonds, and hence results in unnatural conformation, failing to achieve its folding successfully. However, for an efficient folding, the active site in the a′ domain of PDI perhaps assists to escape from this obstacle due to its flexible conformations. It is precisely because of the presence and assistance of these two cysteine residues that hPDI can reduce the nonnative disulfide bonds that have been captured, and then reoxidize the substrate to ultimately form natural disulfide.

To achieve continuous catalysis of the formation of substrate disulfide bond, PDI needs to be regenerated after its disulfide has been transferred to the unfolded substrates. Such reactions involve the interaction between PDI and the Ero1 family enzymes, which present as one isoform (Ero1p) in yeast and two genes encoding Ero1α and Ero1β proteins in higher eukaryotes [98,99,100]. A mechanism for PDI regeneration driven by Ero1 has been proposed as shown in Figure 3, which was elucidated over the past decades [101,102]. The Ero1/PDI oxidative folding pathway uses oxygen (O_2_) as an electron acceptor, producing a byproduct hydrogen peroxide (H_2_O_2_) [103]. Ero1α preferentially oxidizes the a′ domain of reduced hPDI over the a domain, and interacts with the b′ domain of hPDI (with amino acid residues Phe240, Phe249, and Phe304) [104]. Typically, Ero1 utilizes flavin adenine dinucleotide (FAD) as a cofactor, and the oxidizing equivalents transfer from FAD to PDI. Ero1α possesses four regulatory cysteines (Cys94, Cys99, Cys104, and Cys131), which determine its overall activity. Inactive Ero1α (Ox2), containing two disulfide bonds, Cys94/Cys131 and Cys99/Cys104, donates a disulfide bond to hPDI [105,106]. During the redox interaction with hPDI, Cys94 is involved in a direct interaction with hPDI, and likely forms mixed disulfides with hPDI [107]. The disulfide bond Cys94/Cys99 will form and function as a shuttle disulfide. Thus, there is an active-site disulfide bond (Cys394/Cys397) near the FAD isoalloxazine ring, and the Cys94/Cys99 pair transfers oxidizing equivalents from FAD to hPDI. The inner active site, in turn, has been re-oxidized via FAD-mediated electron transfer to O_2_, allowing the cycle to restart [100]. Due to the generation of H_2_O_2_ from Ero1-mediated hPDI oxidation, Ero1 oxidase activity is strictly regulated by the reduction of reduced hPDI to avoid the overoxidation of ER. Such regulation is achieved via noncatalytic isomerization/reduction, in which hPDI plays a critical role [101,108]. However, if the ER is peroxidized, Ero1 will be “inactivated” by auto-reoxidation or oxidizing reduced hPDI to avoid the generation of excessive H_2_O_2_, leading to an ineffective oxidation cycle. Moreover, the oxidative protein folding relay formed by Ero1-PDI can be inactivated by unfolded proteins and folding intermediates when their levels exceed the folding capacity of the system [109].

## 6. Formation of Gluten Network Catalyzed by wPDI

Plant PDI exhibits different functional properties compared with mammalian PDI. For example, soybean GmPDIL-3a and GmPDIL-3b contain nonclassical redox center motif CXXC/S and have no redox activity and molecular chaperone activity in vitro [110]. The chaperone function of plant PDI has already been measured by biochemical assays [110,111,112]. There were also systematic identification and analysis of PDI and PDI-like gene families in different plants, such as rice, wheat, and barley, etc. [34,35,113]. In this section, we will discuss wPDI and the proposed catalytic mechanism with glutenin in dough [52,114].

### 6.1. GMP and wPDI in Wheat Flour

Wheat seeds contain large quantity of proteins, including globulins, albumins, and glutens. The gluten protein, mainly including gliadin and glutenin, is synthesized and accumulated in wheat endosperm during seed development. Glutenin is classified into high molecular weight subunits (HMW-GSs) and low molecular weight subunits (LMW-GSs) based on their molecular weight, which endow the viscoelasticity to dough [115]. Typically, there are four to seven conservative cysteine residues in HMW-GS, and six to eight conservative cysteine residues in LMW-GS. The majority of these cysteine residues form intra-chain disulfide bonds, while some of them form interchain disulfide bonds to yield GMP [116,117,118,119]. GMP is one of the important factors determining the rheological properties of dough [120]. Two reactions play crucial roles in the network formation of GMP among many reactions that present in the dough. One is oxidation, in which free sulfhydryl groups are oxidized into disulfide bonds, resulting in the formation of large protein assembly [121]. The other is the covalent link between glutenins through the sulfhydryl groups (SH)/disulfides (SS) exchange mechanism, which involves the cleavage or reconstruction of the SS bond mediated by the SH of glutenin, resulting in the depolymerization and rearrangement of GMP [122]. Herein, factors relating to interchain disulfide exchange reactions, such as some wheat enzymes involved in disulfide shuffling, should be paid much attention.

**Figure 4:**
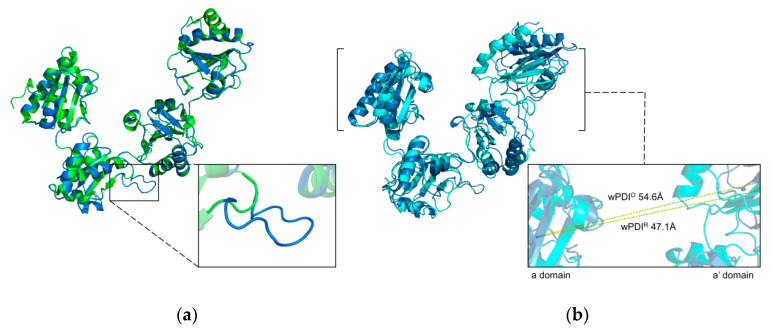
Simulated structure of wPDI. (**a**) Alignment of simulated structure of oxidized wPDI (blue) and experimentally determined structure of oxidized hPDI (green, PDB ID: 4EL1). The enlargement displays the extra loop of oxidized wPDI compared with oxidized hPDI. (**b**) Alignment of the simulated structure of oxidized wPDI (blue) and simulated structure of reduced wPDI (cyan). The enlargement shows the difference of the centroid distances between a domain and a′ domain of both structures, i.e., 54.6 Å for oxidized wPDI (wPDI^O^) and 47.1 Å for reduced wPDI (wPDI^R^).

Many researches on wheat PDI focused on their bioinformatics analysis or application, and the structure of PDI is rarely reported in wheat compared with that in human (Figure 4) [52]. A previous study indicated the presence of wPDI protein in the ER of wheat endosperm by immunolabeling electron microscopy for subcellular localization [123]. Later on, three PDI gene sequences located on 4A, 4B, and 4D chromosomes in Chinese Spring wheat and the DNA and cDNA sequences of PDI from the durum wheat were obtained by Ciaffi et al. Sequence analysis showed that the wPDI gene includes 10 exons and nine introns, and the promoter region contains regulatory elements related to endosperm-specific expression [124,125]. The expression of wPDI reaches its highest level in the middle stage of wheat endosperm formation.

### 6.2. Proposed Catalytic Mechanism of wPDI

**Figure 5:**
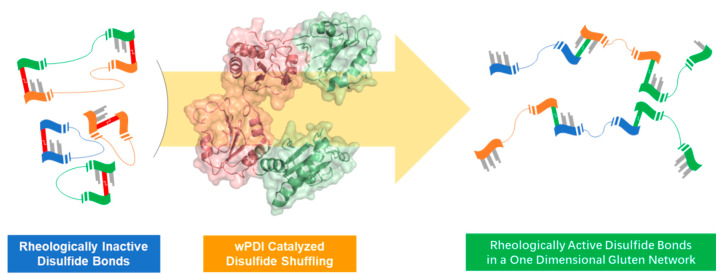
Proposed mechanism of wPDI-catalyzed glutenin macropolymer (GMP) formation. The rheologically inactive disulfide bonds were reduced by wPDI and the active ones were oxidized in between the N- and C- terminals of glutenin subunits to form the gluten network. Left and right: green, orange, and blue ribbons represent different gluten peptides. Grey, red, and green sticks represent free sulfhydryl, rheologically inactive, and active disulfide bonds, respectively. Middle: simulated structure of wPDI, salmon represents its b and b′ domain and pale green represents the a and a′ domain.

Disulfide bonds cross-linking between glutenins is thought to be catalyzed and regulated by wPDI family members during the growth and development of wheat [51,126]. These enzymes mainly exist in the ER of wheat seeds and catalyze the conversion of thiol to disulfide, assisting the formation of intrachain and interchain disulfide bonds of glutens in the wheat ER and ensuring the correct folding and assembly of nascent gluten proteins, as well as resisting various biological and nonbiological stresses [117,118,119]. Studies have shown that the biosynthesis and accumulation of glutenins in developing grains of wheat were closely connected with the expression of wPDI and wPDI-related proteins [115,126,127,128]. Also, several studies have observed that the network structure in GMP could be affected by adding wPDI in vitro, although the conclusions related to the effect of wPDI on dough are not consistent [37,129,130,131]. When cysteines at the two active sites of wPDI were mutated to serine or alkylated, Liu et al. found that these two modified wPDI completely lost the oxidoreductase activity, whereas they still retained the chaperone activity. Furthermore, the gluten matrix would be enhanced by adding these two wPDI [131]. In another study, recombinant wheat Ero1 (wEro1) was expressed and purified in *Escherichia coli*, and the wEro1 was added to wheat dough. By analyzing the rheological properties of the dough, such as viscoelasticity, it was found that wEro1 may promote the cross-linking of disulfide bonds, leading to the formation of gluten polymers [70]. They further investigated the properties of recombinant wEro1 containing FAD cofactor and its influence on Chinese steamed bread qualities. The results demonstrated that wEro1 was capable of catalyzing the reduction of free FAD as well as the oxidation of wPDI, and the denatured protein or GSH were electron donors for wEro1 in catalyzing the oxidation of wPDI. Also, the gluten network could be strengthened after adding wEro1 to Chinese steamed bread during its making process [71]. Recently, Zhao et al. found that wPDI enhanced dough’s alveographic characteristics and bread texture properties by catalyzing the formation of rheologically active disulfide bonds and reduction of inactive ones in a substrate-specific manner (Figure 5) [114]. These facts show that wPDI exhibits a critical effect on GMP.

**Figure 6:**
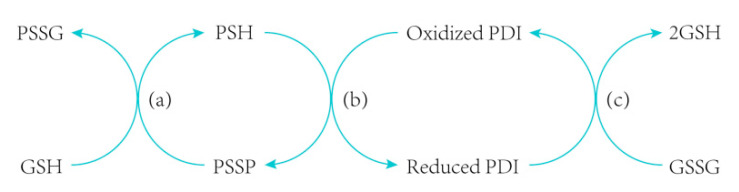
Schematic presentation of gluten disulfide crosslink. Glutathione (GSH) reacts with disulfide bonds within glutenin, allowing the protein to depolymerize (**a**). However, oxidized PDI can catalyze the formation of disulfide bonds in gluten and thus strengthen the gluten (**b**). The reduced PDI produced by the reaction (**b**) can be oxidized by oxidized glutathione (GSSG) to generate GSH (**c**). PSSG represents crosslinked product of GSH and gluten peptide. PSH and PSSP represent sulfhydryl-containing gluten peptide and cross-linked products between glutens, respectively.

wPDI “transfers” disulfide bonds to glutenins, and simultaneously itself is reduced during this catalyzed formation of the gluten network. Meanwhile, to ensure that wPDI could continuously perform the function of catalytic oxidation, the free sulfhydryl in wPDI needs to be reoxidized in parallel. As indicated above, hPDI requires human Ero1 family enzymes to perform the reoxidation step. Such a PDI-Ero1 pathway was also found in higher plants, but the mechanisms appear differently. For example, human Ero1 is a soluble protein, while rice and soybean Ero1 are membrane proteins [132,133,134]. Although Ero1 from different species are all localized in the ER, the underlying mechanisms of their ER retention are not the same—human Ero1 is retained in the ER via covalent interactions with hPDI and ERp44, while rice and soybean Ero1 forms a transmembrane region that is tightly bound to the ER [135]. Even the number of cysteines appears to be highly different between different Ero1 proteins. These may cause a big difference between the PDI-Ero1 pathways of different species. However, since Ero1 family proteins are widely found in eukaryotes, and in wheat it was demonstrated to promote the gluten crosslinking, it could be inferred that wheat employs a similar, though not identical, PDI-Ero1 pathway to aid the reoxidation of wPDI [70]. Namely, Ero1 in wheat ER “transfers” its disulfide bonds to wPDI, and then further shuffles wPDI to the substrate protein to ensure its oxidation activity. In fact, the mechanism of wPDI-catalyzed formation of gluten polymers is complicated, since it is affected by many other factors, including GSH, thioredoxin, and other enzymes, like glucose oxidase and laccase, etc. For example, sulfhydryl in GSH is interchanged with glutenin via an SH/SS exchange reaction, leading to depolymerization of glutenin (Figure 6a), because wPDI and PDI-related proteins family bind to not only thiol-containing glutenin, but also GSH or GSSG (Figure 6b,c) [15,136,137]. In addition, it is known that addition of glucose oxidase improves the dough’s rheological properties [138]. Adding laccase, which catalyzes the cross-linking reaction in gluten, also changes dough rheological properties and thereby improves the dough properties [139]. However, dough rheological properties could be greatly affected by these different factors, which similarly achieve their function through indirectly impacting the sulfhydryl and disulfide bonds among glutenins. In other words, factors affecting the formation of disulfide bonds, particularly wPDI, play a dominant role in gluten polymerization.

## 7. Other Functions of PDI

PDI is mainly used to facilitate nascent polypeptide folding in the ER, but its presence has also been reported at other intracellular and extracellular locations [140,141]. On the aspect of interacting with proteins, evidence was provided to indicate that PDI serves as a subunit of prolyl hydroxylase and triglyceride transferase to stabilize these complexes and retain them in the ER [142,143]. Analogously, in order to prevent procollagen chains from being secreted out of ER before they fold into their natural conformation, PDI interacts with them in the early stages of their assembly [144]. Upon phorbol ester stimulation, PDI is mobilized to the cytosol fraction of human neutrophils, where it associates with a cytosolic regulatory subunit p47^phox^ [145]. This suggests a possible role of PDI as the regulator of anti-inflammatory reaction via neutrophils in human body. On the other hand, because of its ability to activate protein folding, PDI also helps the growth of cancer cells, which requires increased protein synthesis [146]. Drug discovery studies targeting PDI were performed to design inhibitors of the growth of cancer cell lines such, as glioblastoma [147]. Moreover, the complex produced by the combination of PDI and ubiquilin has critical functions as regulatory proteins for CHOP (a CCAAT/enhancer binding protein homolog) mediated cell death [148]. Up-regulation of PDI together with ubiquilin may enhance the tolerance to ischemic stress in glial cells. For other functions, an experiment about ADAM-17, a disintegrin and metalloproteinase, has shown that PDI could be responsible for regulating disulfide(s) in the noncatalytic site of ADAM-17 by exerting its isomerase activity [149]. Additionally, PDI was found to be a neuroprotective protein. A previous study showed that the upregulation of PDI could increase the viability of neuroblastoma cells in the ischemic rat brain [150]. Another study demonstrated that the silencing of PDI can cause increased apoptosis in neuron cells under ER stress [151]. Likewise, nitric oxide (NO) is also essential for the brain′s physiological function, but excessive amounts of NO may lead to brain dysfunction, and this was found to be attributed to the S-nitrosylation effects [152]. Such trans-nitrosation reactions mediate NO internalization from extracellular S-nitrosothiols, and hence inhibit PDI isomerase activity [153,154,155]. It was shown that this NO-induced PDI S-nitrosylation was associated with neurodegenerative disorders, such as Alzheimer′s disease and Parkinson′s disease [155,156,157].

Intriguingly, previous work has shown that PDI could break the disulfide bond within cholera toxin (CT) holotoxin and free its A1 subunit, due to which PDI was reckoned to possess the “unfoldase activity” [158]. However, Cherubin et al. found that such “unfoldase” of PDI doesn′t occur during CT depolymerization, and can′t be interpreted as parts of the enzyme activity of PDI [159]. In short, PDI has a variety of functions, some of which are now commonsense, while others need further elucidation.

## 8. Conclusions

As described above, PDI is a multifunctional protein participating in a variety of redox-related intracellular and extracellular events. It regulates the biological processes, such as protein folding, signal transmission, and cell communication etc., involving the interaction between PDI and substrate proteins. Normally, protein cross-linking begins from the change of protein conformation, which subsequently bridges the cysteines via disulfide bonds, and finally ends up with the formation of interchain disulfide cross-links. The second one is the most critical among these steps. This article mainly reviewed how PDI mediates the formation and isomerization of disulfide bonds during protein folding, especially the newly proposed mechanism of wPDI-catalyzed formation of glutenin networks. Collaboration of PDI family members is needed for completing both folding and maturation of large number of proteins in the ER. However, specific division of labor of PDI family members in the cell is not yet fully clarified. They presumably exhibit either a certain degree of versatility or substrate preferences. The protein cross-linking reaction catalyzed by PDI is complex, and it is necessary to comprehensively use various methods for further in-depth studies. Although much remains unclear, protein folding and related disulfide bonds formation/isomerization catalyzed by PDI are of great significance, not only to related health issues, but also to many other biological molecular assemblies related in other aspects.

## Data Availability

No new data were created or analyzed in this study. Data sharing is not applicable to this article.
